# Preparation of Hierarchical Highly Ordered Porous Films of Brominated Poly(phenylene oxide) and Hydrophilic SiO_2_/C Membrane via the Breath Figure Method

**DOI:** 10.3390/ma11040481

**Published:** 2018-03-23

**Authors:** Hua Yuan, Bing Yu, Hailin Cong, Ming Chi, Yuanzhe Cheng, Chunxin Lv

**Affiliations:** 1 Institute of Biomedical Materials and Engineering, College of Materials Science and Engineering, Qingdao University, Qingdao 266071, China; yuanhua9696@163.com (H.Y.); yubingqdu@yahoo.com (B.Y.); mingchiqdu@163.com (M.C.); yuanzhecheng2014@163.com (Y.C.); chunxinlv18@163.com (C.L.); 2 Laboratory for New Fiber Materials and Modern Textile, Growing Base for State Key Laboratory, College of Chemistry and Chemical Engineering, Qingdao University, Qingdao 266071, China

**Keywords:** SiO_2_ nanoparticles, breath figure method, ordered porous membrane, ice substrate

## Abstract

Porous permeable films materials have very broad prospects in the treatment of sludge-containing waste water due to their large surface area and good microfiltration. In this work, highly ordered porous membranes have been prepared successfully on ice substrates using a poly(phenylene oxide) (BPPO)-SiO_2_ nanoparticle (NP) mixture by the breath figure method. Based on the theory of Pickering emulsion system and capillary flow, particle assisted membrane formation was analyzed. Another two sorts of new membranes SiO_2_/C membrane and hierarchical porous polymer (HPP) membrane, which were obtained by modification of the BPPO-SiO_2_ membrane by calcination and etching, were set up in a further study. Their properties were investigated through the methods of scanning electron microscopy (SEM), fourier transform infrared spectrometry (FTIR), ultraviolet spectrum (UV), capillary electrophoresis (CE), contact angle, and water flux tests. All these results demonstrate that both surface hydrophilicity and fouling resistance of the membrane would be improved by using SiO_2_ as a filler. The membranes with high permeability and antifouling properties were used for microfiltration applications.

## 1. Introduction

In recent years, highly ordered permeable films have attracted great attention in many areas such as chemical, biology, life science and material sciences [1,2,3,4,5,6,7], due to their large surface area and exclusion properties. There are a lot of techniques to synthesize these films, such as micro/nano machining, colloidal crystal template, molecular layer deposition techniques, and so on; however, some of these methods have disadvantages, for example, they are expensive, and unable to control the pore size dynamically [8]. Recently, the breath figure (BF) technique [9,10,11,12] has developed rapidly and has been widely used in preparing order membranes. Furthermore, enhancing the porosity of the membrane is assumed to yield a better performance in terms of permeability, antifouling and solute rejection properties.

In a typical procedure, the BF is caused by to the evaporation of the solvent, which makes the surface temperature of the polymer solution decline in humid conditions, and the water vapor in air condenses into water droplets in the gas-liquid interface. In the process, water droplets condense largely on the surface of the rapid evaporation liquid, and they are surrounded by organic solvent and “suspended” on the top surface. It can thus be considered that a stage of water-in-oil emulsion system is formed. With the evaporation of the solvent, under the action of gravity, Marangoni convection and thermo-capillary force, water droplets were immersed in the solution. When the solvent evaporates completely and water droplets are evaporating continuously, then holes are formed and the films’ structure becomes more ordered [13].

Traditional preparation of highly ordered permeable films by breath figure technique utilizes the pure polymer or mixture of several polymers whose applications are unitary [14,15]. Chen and co-workers fabricated supramolecular porous microstructures by a static breath figure process [16]. Inorganic porous films have been fabricated by the BF technique using polymers containing inorganic precursors and mixing of inorganic particles with polymer solutions [17,18,19,20,21,22]. For instance, Böker et al. prepared a honeycomb film containing CdSe nanoparticles (NPs) [23]. Zhang et al. prepared organic/inorganic hybrid honeycomb films from a block copolymer [24]. Numerous sorts of inorganic NPs have been reported, and many of them are commercially available. Do Sung Huh et al. prepared poly(ε-caprolactone)-graphene oxide honeycomb-patterned films to improve the thermal properties and those films could be used in tissue engineering, sensors, drug delivery, energy storage, etc. [25].

In fact, the behavior of colloidal particles on the liquid-liquid interface has been studied for over a century. The emulsion system is called “Pickering emulsion” in which solid particles are used as emulsion stabilizers [26]. Some researchers have tried to prepare hybrid honeycomb porous membranes by introducing solid nano-particles into the water droplet template. They also tried to prepare composite material for which the inner wall of polymer porous membranes is selectively enriched with quantum point hybrid, using the spontaneous migration of nano-particles at the water-oil interface [27]. However, in this system, the authors neither investigated the effect of particle-stabilized droplets, nor studied the effect of Pickering’s emulsification on breath figure methods.

In this work, BPPO and silica particles are used as model materials to study the auxiliary effect of solid particles in the preparation of ordered porous membrane, and based on Pickering emulsification theory, to discuss the influence of the physical particles in the self-assembly process of breath figure method. Finally, a new method for a particle-assisted water droplet template was established. The performance of the hybrid microfiltration membrane in water treatment was also studied.

## 2. Results and Discussion

### 2.1. Morphology of Microfiltration Membrane

**Figure 1:**
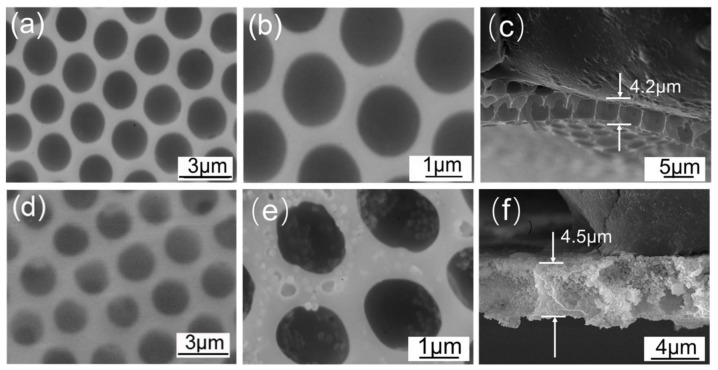
SEM images of BPPO ordered porous membranes prepared on ice substrate using the breath figure method. (**a**–**c**) without SiO_2_-NPs; (**d**–**f**) containing SiO_2_-NPs.

Figure 1a–c shows the SEM images of pure BPPO porous membranes formed under ice substrates by the breath figure method at an environmental temperature 25 °C, humidity of 95%, respectively. The membranes had regular porous structures after being peeled off from the substrates. The surface of pure BPPO membrane was smooth and had honeycomb-type highly ordered porous structures with pore size of approximately 1.8 ± 0.2 μm and a thickness of 4.2 ± 0.3 μm. The low temperature ice surface might hinder the solvent evaporation and speed up condensation of water droplets, which helps the water droplets run through the polymer solutions under the action of gravity, capillary force and Marangoni effect. Therefore, the BPPO membrane was shown to be highly permeable.

A similar study was also performed for BPPO-SiO_2_ membranes on ice substrates via breath figure method under the same conditions of pure BPPO membranes. Ordering and uniformity of the membrane was affected markedly by humidity, solution concentration and nanoparticle size. The SEM image of the BPPO-SiO_2_ membrane (Figure 1d–f) showed ordered micro-sized pores (pore diameter: 1.8 ± 0.3 μm; thickness: 4.5 ± 0.2 μm). The honeycomb-patterned porous films that formed around the template water droplets were prepared successfully and highly ordered. The SiO_2_-NPs were present inside the membrane and were covered with BPPO. The magnified SEM image of the BPPO-SiO_2_ membrane (Figure 1e) shows the BPPO was evenly distributed around the pores and there were SiO_2_ NPs at the pore edges. The weight ratio of SiO_2_ NPs and BPPO added in the CS_2_ was 57.2:42.8, calculated from the weight loss observed in the thermogravimetric curve.

**Figure 2:**
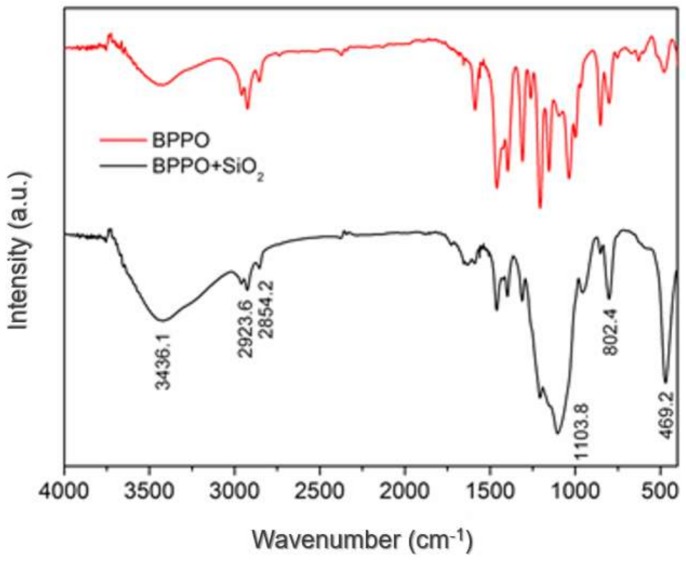
FTIR spectra of BPPO-SiO_2_ membrane and pure BPPO membrane.

Figure 2 shows the fourier transform infrared spectrometry (FTIR) of BPPO-SiO_2_ (black line) membrane and pure BPPO (red line) membrane. In an infrared absorption spectrum of BPPO-SiO_2_, the peaks of –OH, C–H, and C–Br stretching of BPPO and SiO_2_ were observed at 3436.1, 2923.6 and 2854.2, 469.2 cm^−1^, respectively. A characteristic peak was also observed at 802.4 cm^−1^ for benzene displace. At the same time, the fact that the peak of Si-O stretching at 1103.8 cm^−1^ appeared in the black was not observed in the red suggested that SiO_2_ NPs were dispersed in the BPPO polymer successfully and did not affect the structure of the polymer.

### 2.2. Analysis of Particle Assisted Membrane Formation

Under the action of the Pickering emulsification effect, the solid particles can be adsorbed on the water-oil interface spontaneously to reduce the free energy of the interface [28,29,30]. The adsorption phenomena at the interface of colloid particles were firstly described by Pieranski [28]: If a microsphere is transferred from one phase, the energy *E* required to remove from the interface is defined by the Formula (1).

(1) $$E=\pi R^{2}\gamma_{\mathrm{wo}}{\left(1-\cos\varphi \right)}^{2}$$

In which, *R* is the radius of the particle; γ_wo_ is the interfacial tension between water and CS_2_; φ is the three-phase contact angle of these particles at the interface. In addition to the particle appearing extremely hydrophobic (φ = 180°) or extremely hydrophilic (φ = 0°), the particle can always be easily adsorbed at the interface under the driving of decreasing the free energy of the system. Once the particles migrate to the interface, they can achieve a more stable adsorption. From the Formula (1), *E* is proportional to *R*^2^, so for micron and submicron size particles, the energy value of the thermodynamic perturbation is much less than *E*, and desorption of the particles will not spontaneously occur after the adsorption [31,32]. From the Figure 2d, it can be seen that the particles are highly selectively distributed in the porous surface of the membrane; they are almost all in the walls of the pores and none are distributed in the holes. This confirms that the efficiently direct migration of particles occurs after water condensation, and SiO_2_ microspheres with hydrophilic properties are greatly enriched in the liquid-liquid interface formed by the organic solvent and water droplets. Particles are embedded in the inner wall of the polymer matrix cavity after complete volatilization of solvent and water droplets, from Figure 1e. Particles undergo local enrichment in the connected holes and spontaneously create a physical barrier to prevent droplet gathering in the film forming process.

In addition, Brinks found that silica particles can stabilize emulsions by forming dense, close-packed monolayers at the droplet surface [29]. Furthermore, due to strong volatility of CS_2_ and capillary flow, particles showed ring pattern particle assembly arrays in a volatile process [30]. Overall, annular nanoparticle array selectively distributed in order porous substrate is formed by the Pickering emulsion effect and capillary flow together. That is, the process is driven by the forces of thermodynamics and kinetics

### 2.3. SiO_2_/C Membrane and HPP Microfiltration Membrane

**Figure 3:**
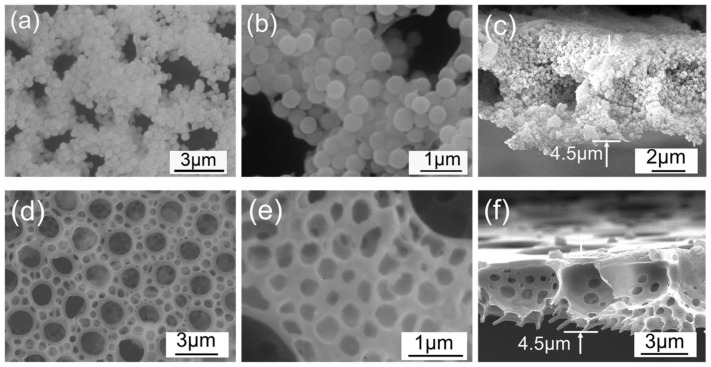
SEM images of the SiO_2_/C membrane (**a**–**c**) and the HPP membrane (**d**–**f**).

**Figure 4:**
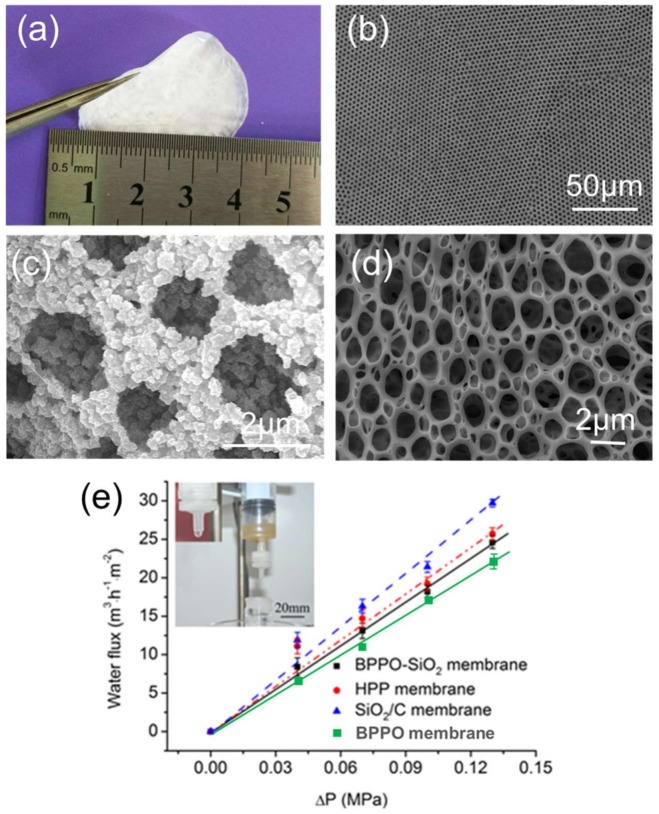
Microfiltration performance of the membranes: (**a**) photo of the BPPO-SiO_2_ membrane; (**b**) SEM image of the BPPO-SiO_2_ membrane; (**c**) SEM image of the SiO_2_/C membrane’s surface after soaking in water for 12 h; (**d**) SEM image of the hierarchical porous polymer (HPP) membrane; (**e**) Relationship between pressure and water flux, inset shows a photo of waste water filtration by the membrane and purified water after the filtration.

The SiO_2_ membrane with amorphous carbon as a binder (SiO_2_/C membrane) comprises the following steps: put the BPPO-SiO_2_ membrane in a laboratory tube furnace to calcine at 600 °C under nitrogen atmosphere for 10 min as shown in Figure 3a–c. The membrane maintained its ordered porous structure, and its magnified picture (Figure 3b) showed that SiO_2_-NPs accumulated closely. To investigate the stability of the SiO_2_/C film, the films were soaked in water for 12 h and it was found that the SiO_2_/C films retained their structures in water, shown in Figure 4c. This result suggested that the NPs were connected at their surfaces by a carbon skeleton. Herein, the method does not need sacrificial templates for preparation of an inorganic membrane with micro sized pores.

Additionally, the BPPO-SiO_2_ membrane was etched by hydrofluoric acid to remove the SiO_2_-NPs and then a hierarchical porous polymer membrane (HPP membrane) with nano- and micro-sized pores was obtained shown in Figure 3d–f. The bigger pores formed by evaporated of water droplets are well-proportioned and the smaller pores are formed by SiO_2_-NPs. These hierarchically structured porous films can be used for a membrane separation because of their high porosity. The thickness of both SiO_2_/C film and HPP membrane is ~4.5 μm ± 0.5 μm.

### 2.4. Application in Microfiltration

These membranes are flexible and robust, which makes them useful for microfiltration applications (Figure 4a). The membranes’ surface SEM images were shown in Figure 4b–d. The membrane is put into a permeation module which is a commercially available product used for sample pretreatment. Moreover, the membranes were not freestanding film and were stand by spunlace non-woven fabric (thickness: 0.3 mm, porosity: 93%). Waste water with a mud concentration of 5 g/L (particle diameter ≥2 μm as measured by dynamic light scattering) was filtrated successfully using the porous membrane shown in the photo inset of Figure 4e, and the filtering effect is obvious. The water flux was 17.0, 18.2, 19.2 and 21.4 m^3^·m^−2^·h^−1^ when the feed pressure is 0.1 MPa for the BPPO membrane, BPPO-SiO_2_ membrane, HPP membrane and SiO_2_/C membrane, respectively, as shown in Figure 4e. This is larger than previously reported [31]. For the HPP membrane and SiO_2_/C membrane, the rate of through-hole greatly increases, so this is perhaps the principal reason why the HPP and SiO_2_/C membranes’ steady water fluxes are higher than BPPO-SiO_2_ membrane. The water flux of the BPPO-SiO_2_ membrane is bigger than that of the BPPO membrane, mainly due to the hydrophilicity of SiO_2_ which can reduce the adsorption of pollutants on the membrane surface. A linear relationship can be seen between pressure and water flux for the four kind membranes, which fits the calculated value very well according to the Hagen–Poiseuille equation [31]. The Hagen–Poiseuille equation assumes that the flow is laminar viscous and incompressible and that travels constantly through the circular cross-section which best fits the circumstances of the microfiltration membranes used in this research. 

To study the effect of SiO_2_ nanoparticles on the filtration of the BPPO-SiO_2_ membrane, membrane water flux attenuation coefficient *m* was tested under the same experimental conditions for the pure BPPO membrane and BPPO-SiO_2_ membrane after water filtration. The *m* is 8.7 and 4.5, respectively. Membrane pollution is largely due to the formation of a gel layer by the interception of impurities on the surface of membrane. Strong hydrophilicity of nano SiO_2_ in the BPPO-SiO_2_ membrane leads to the formation of a layer of water film quickly on the membrane surface, which prevents the formation of the gel layer. Thus, the membrane pollution resistance ability greatly improved. In addition, it shows a remarkable toughening effect and obvious nanometer surface effect, due to mutual penetration of the Si hydroxy group on the SiO_2_ nanoparticles and BPPO, forming a polymer physics junction.

### 2.5. Anti-Fouling Performance

**Figure 5:**
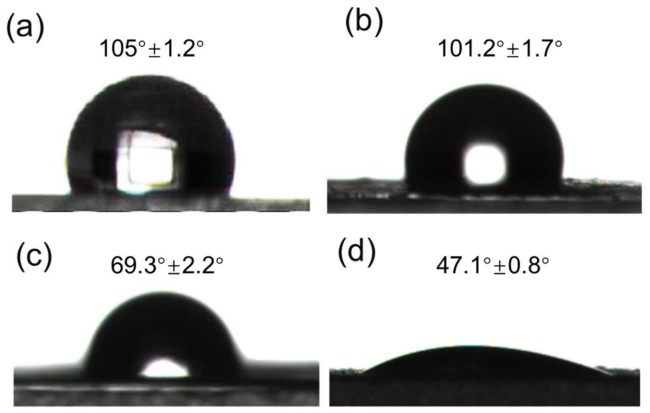
Contact angle of water on the membranes: (**a**) pure BPPO membrane; (**b**) HPP membrane; (**c**) BPPO-SiO_2_ membrane; (**d**) SiO_2_/C membrane.

Membrane fouling in microfiltration is a key factor affecting the economic and technological viabilities of the ultrafiltration processes. It depends on the obtained permeate fluxes and the stability overtime and causes of membrane fouling are quite complex. Membrane surface hydrophilicity is the main factor affecting the surface-adsorption properties of the membranes. Hydrophilicity is associated with the surface adsorption properties of the membrane and improving the hydrophilicity of a membrane can reduce membrane fouling to some extent [32]. The hydrophilicity of four kinds of membranes was researched by testing the contact angle of water. The anti-fouling ability of the BPPO, HPP, BPPO-SiO_2_ and SiO_2_/C membranes was investigated and the contact angle of water was approximately 105°, 101.2°, 69.3° and 47.1° for the BPPO membrane, HPP membrane, BPPO-SiO_2_ membrane and SiO_2_/C membrane, respectively (Figure 5). This shows that hydrophilicity of BPPO membrane could be improved significantly using SiO_2_ as a filling and this result showed that the SiO_2_/C membrane possessed a better hydrophilicity. The Si–OH chains of membrane surface can weaken the hydrophobic interactions between protein molecules and microfiltration membrane and affect the surface adsorption of the protein, although the membrane-surface morphology was altered by an increase of surface “valleys”. Conversely, the permeation-flux increase of the modified membrane was attributed to the surface hydrophilicity and efficient filtration area due to the addition of hydrophilic inorganic SiO_2_ particles. To be specific, the hydrophilic Si–OH chains have better biocompatibility, large excluded volume and unique coordination with surrounding water molecules in protein and sewage aqueous solution. Thus, the sorption and conglomeration of protein was according weakened and the anti-fouling ability improved. 

**Figure 6:**
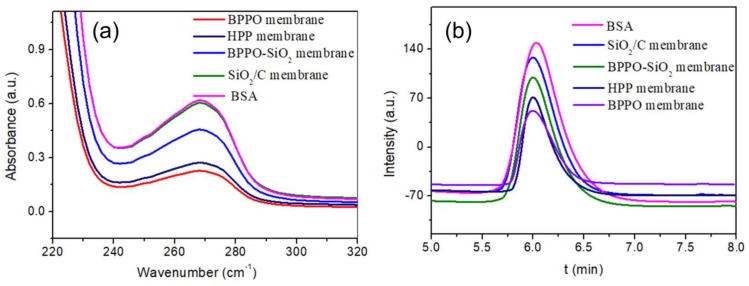
(**a**) UV-vis spectra of BSA in the filtered fluid and (**b**) BSA absorption peak using the bare capillary. Conditions of CE: buffer, 40 mM phosphate (pH = 7.0); applied voltage, +15 kV; UV detection, 214 nm; capillary, 75 μm ID × 50 cm (41 cm effective); capillary temperature, 25 °C.

In addition, the eutrophication of natural water will obstruct the application of membrane technology significantly. It is necessary to research the protein absorption on the membrane [33,34]. Therefore, the antifouling performance of porous membranes is evaluated by the method of dynamic protein absorption and using a BSA protein as an analogue, which is readily available and easy to measure by ultraviolet spectrum (UV) and capillary electrophoresis (CE). The 1L BSA solution (1 g/L, pH 7.0) was used to pass through the filtration membrane ten times at 0.04 MPa. The BSA content in the filtering liquid was tested. Figure 6a shows the UV-vis reflection spectra of the BSA at a wavelength of 230~320 nm and the absorption peak of BSA solution using CE in Figure 6b. The higher the peak value, the higher the protein content in the filtrate and the lower the membrane adsorption. The adsorption amount of BSA clearly decreases with the existence of SiO_2_ for the BPPO-SiO_2_ membrane and SiO_2_/C membrane. The hydrophilic SiO_2_ particles on the membrane surface reduce the protein adsorbed on the membrane surface. This is very meaningful for membranes’ anti-fouling performance. 

## 3. Materials and Methods

### 3.1. Materials

Chloroform, methanol, carbon disulfide, hydrofluoric acid (HF), tetraethoxysilane (TEOS), polyphenylene oxide (PPO, Mw = 40 000) and bromine were analytical grade and purchased from Aldrich (St. Louis, MO, USA). Water was purified by a Millipore system (Milli-Q, Millipore, Darmstadt, Germany). Absolute ethanol and ammonia solution (28 wt. % for ammonia) were obtained from Tieta Chemical Company (Taipei, Taiwan). All chemicals were used as received without further purification. The brominated polyphenylene oxide (BPPO) was prepared using established protocols, based on the method of White and co-workers [35].

### 3.2. The Synthesis of Silica Microspheres

Monodisperse SiO_2_ spheres were prepared using a modified Stöber procedure reported elsewhere [36]. The typical procedure can be described as follows: Solution A is a mixture of 2.08 g TEOS (tetraethyl orthosilicate) and 50 mL EtOH (ethanol), and solution B is a mixture of 3.85 mL of 28 wt. % ammonia, 3.0 mL of water and 40 mL of EtOH. The typical preparation involves rapidly mixing two solutions at room temperature under magnetic stirring. After 24 h, the SiO_2_ particles in the latex were separated by centrifugation at 6000 rpm and were washed with DI water several times. Finally, the purified products were dried at 80 °C for 48 h and dynamic light scattering showed that the average diameter of SiO_2_ nanoparticles was 200 ± 10 nm.

### 3.3. Preparation of Ordered Porous Membranes

**Figure 7:**
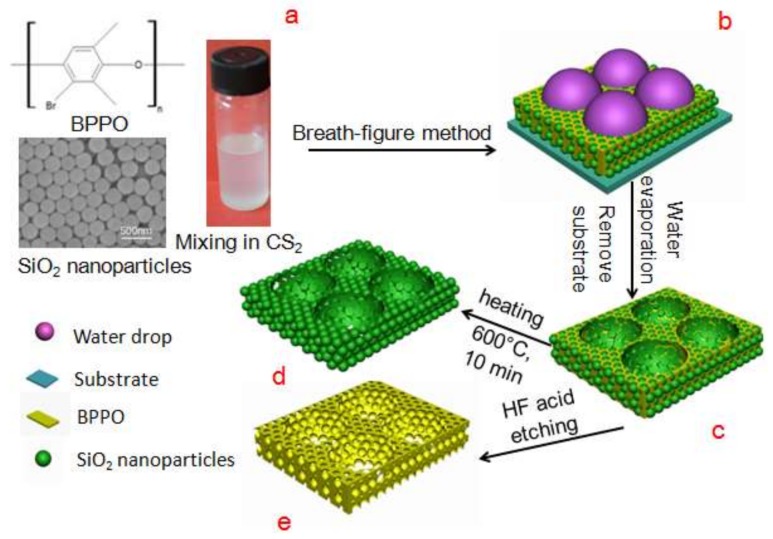
Schematic illustration of the preparation of the hierarchical porous membrane. (**a**) structural formula of BPPO, SEM image of SiO_2_ nanoparticles and casting membrane solution; (**b**) water droplets self-assemble on the membrane surface; (**c**) BPPO-SiO_2_ membrane; (**d**) SiO_2_/C membrane and (**e**) HPP membrane.

As shown in Figure 7, ordered porous membranes containing SiO_2_-NPs were formed by casting BPPO/carbon disulfide (CS_2_) solutions (10 g·L^−1^) with SiO_2_-NPs onto ice substrate under a fixed humidity of 80–90% [31,37]. A typical procedure is as follows: 50 μL of BPPO solution (10 g·L^−1^) containing SiO_2_-NPs (the membrane without SiO_2_ NPs named BPPO membrane) was deposited onto the ice surface, an area of 64 cm^2^ (Figure 7a), and was put into a petri dish covered with a lid, in which the humidity and temperature were well controlled by a wet machine and heater. The water droplets coagulated on the surface of the polymer solution due to high humidity and cooling caused by CS_2_ volatilization; then the water droplets immersed into the solution by gravity, Marangoni convection and thermo-capillary force (Figure 7b). After complete evaporation of the solvent and water drop, the honeycomb-patterned porous membrane containing SiO_2_-NPs was obtained on the ice surface, named the BPPO-SiO_2_ membrane (Figure 7c). The BPPO-SiO_2_ membrane was calcined in tube furnace at 600 °C in the atmosphere of nitrogen for 10 min to obtain the SiO_2_/C membrane (Figure 7d). In addition, by etching the BPPO-SiO_2_ membrane in HF solution to remove SiO_2_-NPs, a hierarchically ordered porous membrane can be directly obtained. This is called the HPP membrane (Figure 7e).

### 3.4. Characterization

Surface morphologies of the obtained membranes and structures were characterized by scanning electron microscopy (SEM, JEOL JSM-6390LV, Japan Electronics Co., Tokyo, Japan), operating at a 20 kV accelerating voltage. FTIR spectrum was measured in a Bruker Alpha type infrared spectrometer, using the KBr compression method; scan range 400~4000 cm^−1^, scanned 64 times and resolution of 4 cm^−1^. The thermo-gravimetric curve of BPPO and BPPO-SiO_2_ NPs were obtained on comprehensive thermal analyzer (Q600 SDT, TA Instruments, New Castle, DE, USA) in nitrogen atmosphere with temperature range of 25~600 °C at a heating rate of 4 °C·min^−1^. The diameter of the SiO_2_ nanoparticles was measured with a light scattering particle diameter measurement instrument (Zetasizer NANO-ZS, Malvern Instruments Co. Ltd., Worcestershire, UK). Wettability of the porous membranes was characterized by an automated contact angle goniometer (JY-82, Dingsheng Testing Equipment Co. Ltd., Chengde, China). The static contact angle of all membranes was recorded 30 s after a water drop was added onto the top surface of the membranes to get a steady reading. To investigate the protein resistance characteristics, static protein absorption was measured as follows: 4 cm^2^ of each membrane was soaked in the 0.5 wt. % BSA/PBS buffer (pH = 7.0) solution (BSA: bovine serum albumin, PBS: phosphate buffered saline) for 24 h at room temperature. To observe the attenuation rate of water flux and antifouling performance, the higher concentration of BSA solution (1 g/L, pH 7.0) was used to pass the solution through the filtration membrane. The *m* was calculated by the following formula: *m* = (J_0_ − J_1_)/J_0_ × 100% (J_0_, initial penetration flux; J_1_, the membrane permeates flux after filtering for 6 h). UV-Vis spectrometer (TU-1810, Beijing Puxi General Instrument Co. Ltd., Beijing, China) at a wavelength of 295 nm was used to research the protein absorption of membranes in this work and using BSA as an analogue. The capillary electrophoresis (CE) experiments were performed on a CL1020 high performance capillary electrophoresis instrument (Huayang Liming Instrument Co., Beijing, China).

## 4. Conclusions

In this paper, highly ordered porous BPPO-SiO_2_ membranes were successfully synthesized on ice substrate using the breath figure method, and particle assisted membrane formation was analyzed based on the theory of Pickering emulsion system and capillary flow. Further, SiO_2_/C membranes were also prepared through calcining the origin membrane in nitrogen atmosphere at 600 °C for 10 min; and HPP membranes were prepared through etching the origin membrane in an HF solution. These three kinds of membranes have nano- to micro-sized porous structures, and all are thermally and chemically stable. Waste water with a mud concentration of 5 g/L was successfully filtrated. The permeation flux increase of the membrane is attributed to the hydrophilic inorganic nano-sized SiO_2_ particles. The pure water flux changes indicated the filtration performance improvement for all four kinds of membranes. The addition of SiO_2_ apparently improved the hydrophilicity, which helped to enhance the anti-fouling ability of the membrane.
